# Unfractionated heparin improves the clinical efficacy in adult sepsis patients: a systematic review and meta-analysis

**DOI:** 10.1186/s12871-021-01545-w

**Published:** 2022-01-21

**Authors:** Sifeng Fu, Sihan Yu, Liang Wang, Xiaochun Ma, Xu Li

**Affiliations:** Department of Critical Care Medicine, the First Affiliated Hospital of China Medical University, North Nanjing Street 155, Shenyang, 110000 Liaoning Province China

**Keywords:** Anticoagulant treatment, Meta-analysis, Sepsis, Septic shock, Unfractionated heparin

## Abstract

**Background:**

The anticoagulant treatment and clinical efficacy of heparin in sepsis remains controversial. We conducted a meta-analysis to estimate the clinical efficacy of unfractionated heparin (UFH) in adult septic patients.

**Method:**

A systematic review of Medline, Cochrane Library, PubMed, Embase, WEIPU database, CNKI database, WANFANG database was performed from inception to January 2021. We included Randomized controlled trials (RCTs) and the main outcome was 28 d mortality. Data analysis was performed with Review Manager (RevMan) version 5.3 software. The meta-analysis included 2617 patients from 15 RCTs.

**Results:**

Comparing to control group, UFH could reduce 28 d mortality (RR: 0.82; 95% CI: 0.72 to 0.94) especially for patient with Acute Physiology and Chronic Health Evaluation II (APACHE II) > 15, (RR: 0.83; 95% CI: 0.72 to 0.96). In UFH group, the platelet (PLT) (MD: 9.18; 95% CI: 0.68 to 17.68) was higher, the activated partial thromboplastin time (APTT) was shorter (MD: -8.01; 95% CI: − 13.84 to − 2.18) and the prothrombin time (PT) results (*P* > 0.05) failed to reach statistical significance. UFH decreased multiple organ dysfunction syndrome (MODS) incidence (RR: 0.61; 95% CI: 0.45 to 0.84), length of stay (LOS) in ICU (MD: -4.94; 95% CI: − 6.89 to − 2.99) and ventilation time (MD: -3.01; 95% CI: − 4.0 to − 2.02). And UFH had no adverse impact on bleeding (RR: 1.10; 95% CI: 0.54 to 2.23).

**Conclusion:**

This meta-analysis suggests that UFH may reduce 28 d mortality and improve the clinical efficacy in sepsis patients without bleeding adverse effect.

**Supplementary Information:**

The online version contains supplementary material available at 10.1186/s12871-021-01545-w.

## Background

Sepsis is defined as life-threatening organ dysfunction by a dysregulated host response to infection [1]. The pathophysiology of sepsis is complicated, involving both pro-inflammatory and anti-inflammatory pathways, as well as a whole array of immunological and non-immunological mechanisms [2], such as the coagulation system and the neuroendocrine system [3].

Many research studies focused on the possibility that anticoagulant therapy could improve the mortality of sepsis patients. However, there is a considerable controversy regarding the treatment of coagulopathy in sepsis. There is positive results in Yamakawa et al’s group which meant anticoagulant therapy was associated with better outcome according to the deterioration of both DIC and disease severity for septic patients [4]. Whereas, there were negative results in Walkey et al’s team that among patients with Atrial Fibrillation during sepsis, parenteral anticoagulation was not associated with reduced risk of ischemic stroke and was associated with higher bleeding rates [5]. There is no anticoagulant therapy has been proven effective, which is probably due to the importance of coagulation activation in host defense mechanisms during sepsis [6]. Thus, it’s difficulty to choose the appropriate target, the right timing, and the adequate dose.

Heparin is a widely used anticoagulant which can prevent venous thromboembolism (VTE). In 2016, Surviving Sepsis Campaign (SSC) guideline first evaluated the use of heparin for the treatment of sepsis and septic shock [7]. The Japanese guideline for management of sepsis was against the use of heparin or heparin analogs as a standard treatment in sepsis-associated DIC [8]. The World Health Organization (WHO) recommended heparin use in critically ill patients with COVID-19 to prevent thromboembolism in 2020 [9]. All of these indicated that anticoagulant therapy has attracted worldwide attention and is still controversial. The role of heparin in sepsis still needs to be verified by randomized controlled trials (RCTs).

Heparin contains UFH and low molecular weight heparin (LMWH). The mechanisms of action for UFH and LMWH are different in sepsis, so their effects are various. UFH mainly inhibits the activity of thrombin and factor (F) Xa by binding to antithrombin (AT). The inhibition of thrombin requires a heparin chain comprising of at least 18 saccharide units. Therefore, LMWH can only exert anticoagulant effect by inhibiting the action of FXa. Furthermore, recent research studies proved that UFH possesses various biological properties, such as anti-inflammatory and immunomodulatory effects [10].

UFH seems to be more promising in the treatment of sepsis because of its multiple biological activities. Our previous studies have shown that UFH could protect endothelial cells, improve endothelial barrier dysfunction, and inhibit inflammatory response to preserve organ function and improve the prognosis of septic patients [11, 12].Therefore, RCTs were included in our study that researched the function of UFH in sepsis.. Although three meta-analysis have discussed the effect of heparin in sepsis, none of them highlighted the UFH application in clinical practice [13–15]. Therefore, this article aimed to assess the clinical efficacy of UFH in adult patients with sepsis, septic shock, or DIC.

## Methods

### Search strategy

The research was in accordance with the Preferred Reporting Items for the Systematic Reviews and Meta-Analyses (PRISMA) guidelines [16]. We conducted electronic databases screening including Medline, Cochrane Library, PubMed, Embase, WEIPU database, CNKI database, WANFANG database from inception to January 2021. The controlled vocabulary came from Medical Subject Headings (MeSH) in PubMed and Chinese Medical Subject Headings (CMeSH) in SinoMed.

For example, “Sepsis” [MeSH], “DIC” [MeSH], “Heparin” [MeSH] and corresponding keywords were used for search with various combinations of the operators “AND” and “OR” in PubMed. The search was slightly adjusted according to the requirements of the various databases. The PubMed strategy details were presented in the Supplementary Table S1. At the same time, we manually searched the RCTs, meta-analyses, and systematic reviews for studies that were missed in the initial electronic search. There was no restriction on language or year of publication. The searching was duplicated and the last search update was January 2021. Furthermore, a third reviewer intervened whenever there was a disagreement.

### Inclusion criteria

Studies that were included met the following criteria:1. RCTs; 2. Studies that aimed to assess the clinical efficacy of UFH treatment on mortality, the incidence of bleeding complications and the coagulation indicators, such as the platelet (PLT), prothrombin time (PT) and activated partial thromboplastin time (APTT); 3. Participants in studies were diagnosed with sepsis, septic shock or DIC; 4. The administration of low-dose UFH, given at any route or frequency.

### Exclusion criteria

1.Quasi-randomized controlled trials, for example randomization according to hospital number; 2.Review, repeated literature reports, or animal studies; 3. Studies involving pediatric patients, pregnant or lactating patients; 4.Ex-transplant recipients, patients who had been treated with anticoagulant drugs 48 h ago and had coagulation disorders or history of abnormal coagulation; 5.Patients’ PLT < 30 × 10^11^/L; 6.Patients with severe brain trauma, cerebral aneurysm, arteriovenous malformation and gastrointestinal bleeding history; 7.Renal failure required hemodialysis (hemodialysis) or hemofiltration (hemofiltration); 8.Both the experimental and control group received UFH.

### Data extraction

Two researchers independently extracted the data from each article that met the inclusion criteria but not exclusion criteria. If the opinion of two reviewers conflicted in the process, another reviewer evaluated the studies independently. We then finalized the decisions through group discussion. The following data were extracted: the first author’s name, year of publication, number of study sites, number of patients, population, age, Acute Physiology and Chronic Health Evaluation II (APACHE II) score, intervention (dose and duration), control treatment, the 28 d mortality and incidence of bleeding complications.

### Outcomes

The primary outcome was 28 d mortality. We made reference to APACHE II score to perform subgroup analysis. Also, PLT, PT and APTT were regarded as the coagulation indicators**.** Multiple organ dysfunction syndrome (MODS) incidence, length of stay (LOS) and the duration of ventilation in ICU were recorded as the secondary outcomes. We graphically displayed the outcomes by forest plots and visually inspected the potential publication bias with a funnel plot [17].

### Assessment of risk of bias

The Cochrane Collaboration Risk Of Bias tool (ROB) was utilized to assess the quality of all analyzed trials by two authors [18]. Disagreements between the reviewers were resolved by negotiating with another author. ROB estimated the selection bias by random sequence generation and allocation concealment, performance bias by blinding of participants and personnel, detection bias by blinding of outcome assessment, attrition bias by incomplete outcome data, reporting bias by selective reporting and other potential sources of bias [18]. Each domain was referred to as “low risk,” “high risk,” or “unclear risk” which was identified depended on the researcher’ response [18]. The overall risk of bias for the result was the least favourable assessment across the domains of bias [18].

### Statistical analysis

The meta-analysis was performed using the Review Manager (RevMan) version 5.3 software. Reference to Cochrane Handbook of Systematic Reviews, 28 d Mortality and bleeding complication (Binary variables) were expressed as relative risk (RR) with 95% confidence intervals (CIs), a weighted pooled RR was calculated using the Mantel-Haenszel method [19]. The continuous variables were expressed as mean differences (MD) with 95% CIs, for example, MODS incidence and LOS in ICU [20]. Jaimes et al’ recorded the APACHE II score as median and interquartile range (IQR), We calculated its mean and standard deviation according to the sample size with a calculator [20].

I^2^ test was used to measure statistical heterogeneity. When I^2^ = 0, there was no heterogeneity. When the I^2^ < 40% or I^2^ between 30 and 60%, there was considered as low or moderate statistical heterogeneity in the studies, the fixed effect model was used for analysis [19]. Random effects model was used to analyze studies which had an I^2^ > 50% [19]. All results were summarized in forest plots with both point estimates and alpha = 0.05 displayed.

### Evidence grade

Two researchers graded the evidence for each outcome based on the following six domains: study design, risk of bias, inconsistency, indirectness, imprecision, and publication bias. If there were discrepancies, another author would take part in this discussion. The strength of evidence could be classified as “high,” “moderate,” or “low” [21].

## Results

### Study selection

**Fig. 1:**
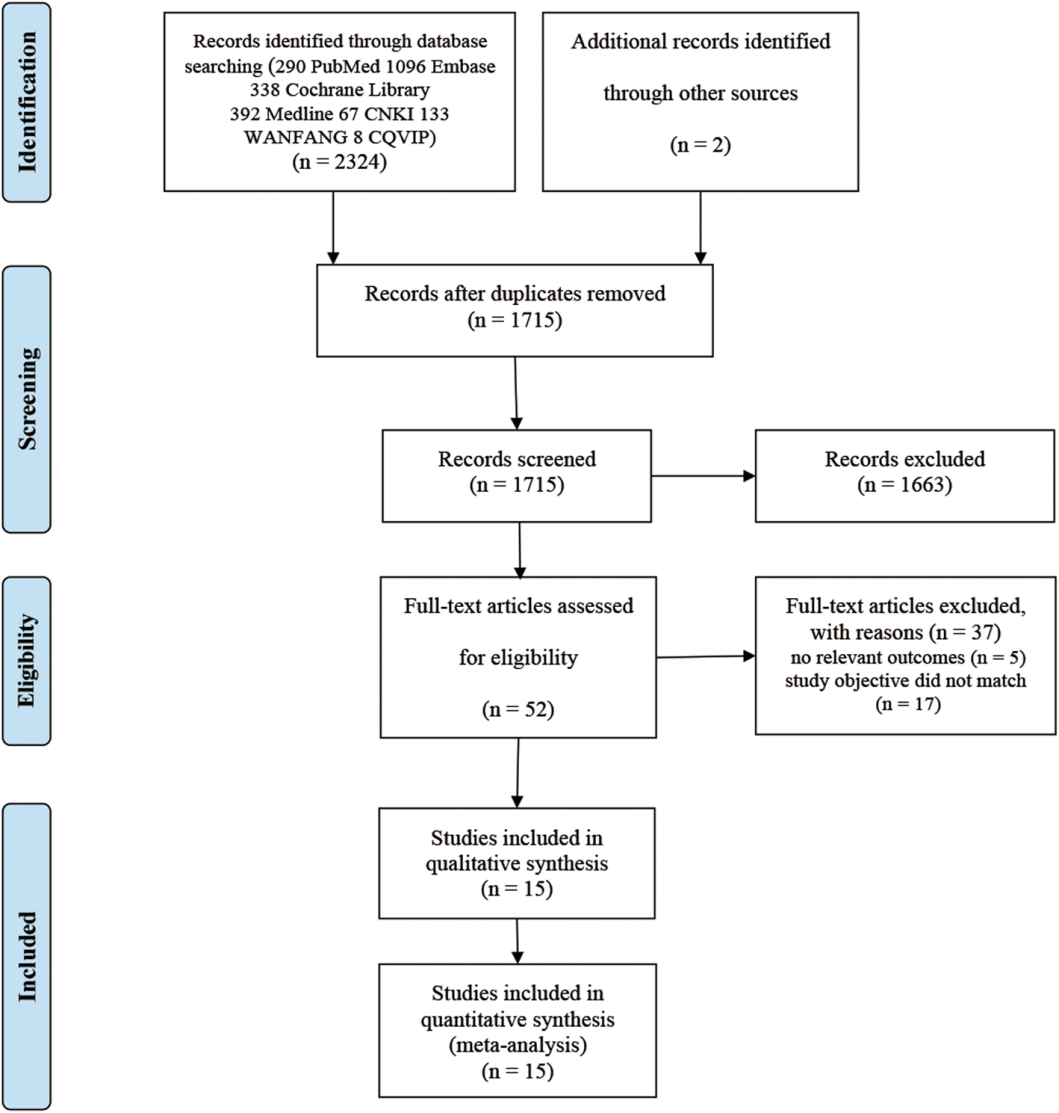
The meta-analysis flowchart

Of the 2326 citations identified from electronic and hand searches, after duplicate checking and screening the titles and abstracts of all searched articles, we selected 52 studies for full text review. Through the full-text screening, this meta-analysis contained 15 studies [22–36], as the flowchart shown (Fig. 1 and details seen in the supplementary Table S2). During searching, both English and non-English articles were considered, thus 11 Chinese articles and 4 English articles were included.

### Study characteristics

The characteristics of the included studies were shown in Tables 1, 2. All the RCTs totally reported 2617 participants, in which 1079 patients received UFH as experimental group and 1538 patients received placebo (saline) or usual care as control group. Except for a lack of 28 d mortality data in Zhang et al. and Guo et al’ study [24, 36], the remaining 13 studies all performed analyses of 28 d mortality. Zhang et al’s study didn’t report APACHE II score [33].

**Table 1:** The basic characteristics of the included studies

Article, year	Number of researches centers	Total (n), UFH/Control	Population	Age (Yr)		*P* value
				UFH	Control	
Deng et al, 2017 [22]	1	118, 62/56	Sepsis and septic shock	62.5 ± 13.9	61.5 ± 15.2	*P* > 0.05
Gu et al, 2014 [23]	1	42, 21/21	Sever sepsis	64.6 ± 12.7	69.7 ± 16.3	*P* > 0.05
Guo et al, 2019 [24]	1	90,45/45	Sepsis with pre-DIC	64.75 ± 7.88	68.87 ± 6.16	*P* > 0.05
Hou et al, 2011 [25]	1	40, 20/20	Sever sepsis	58.6 ± 15.4	57.7 ± 12.6	*P* > 0.05
Jaimes et al, 2009 [26]	1	319, 159/160	Sepsis	57 (IQR: 39–70)	55 (IQR: 40–72)	*P* > 0.05
Levi et al, 2007 [27]	224	1440, 485/955	Sever sepsis	59.6 ± 16.1	58.4 ± 16.0	*P* = 0.90 > 0.05
Liu et al, 2014 [28]	1	37, 22/15	Sepsis	48.86 ± 14.3	47.47 ± 14.68	*P* = 0.935 > 0.05
Liu et al, 2009 [29]	1	27, 12/15	Sepsis	47.4 ± 16.8	48.3 ± 14.1	*P* = 0.708 > 0.05
Peng et al, 2013 [30]	1	112, 56/56	Sepsis with pre-DIC	63.5 ± 7.8	63.5 ± 7.8	*P* > 0.05
Wang et al, 2012 [31]	1	48, 24/24	Sepsis	52.7 ± 9.4	54.3 ± 8.6	*P* > 0.05
Wu et al*,* 2011 [32]	1	85, 45/40	Sepsis	68 ± 13	67 ± 14	*P* = 0.684 > 0.05
Zhang et al, 2006 [33]	1	22, 11/11	Sever sepsis	59.55 ± 6.15	59.36 ± 8.05	*P* > 0.05
Zhao et al, 2009 [34]	1	79, 37/42	Sepsis	61.5 ± 11.9	60.6 ± 15.9	*P* > 0.05
Zhao et al, 2007 [35]	1	52, 27/25	Sever sepsis	NR	NR	*P* > 0.05
Zhang et al*,* 2016 [36]	1	106, 53/53	Sepsis	43.5 ± 6.6	43.5 ± 6.6	*P* > 0.05

*NR* not reported, *UFH* unfractionated heparin, *IQR* interquartile range

**Table 2:** Characteristics and physiological parameters of patients in the included studies

Article, year	APACHE II score		*P* value	Intervention		28 d Mortality (%)		*P* value
	UFH	Control		UFH	Control	UFH	Control	
Deng et al, 2017 [22]	20.5 ± 4.2	21.8 ± 6.2	*P >* 0.05	UFH 7d (vein)	Usual care	21.7	30.9	*P* < 0.05
Gu et al, 2014 [23]	12 ± 9	16 ± 5	*P >* 0.05	UFH 5–10 U/(kg*h) 7d (vein)	Usual care	23.8	33.3	*P* > 0.05
Guo et al, 2019 [24]	15.22 ± 5.34	18.16 ± 5.53	*P* > 0.05	UFH 70 U/kg 5-7d (vein)	Usual care + saline	NR	NR	NR
Hou et al, 2011 [25]	11.8 ± 3.5	15.3 ± 6.4	*P* > 0.05	UFH 3-4 U/kg/h 7d (vein)	Usual care	15	25	*P* = 0.429 > 0.05
Jaimes et al, 2009 [26]	9 (IQR: 7–13)	10 (IQR: 6–14)	*P* > 0.05	UFH 500 U/h 5-7d (vein)	Usual care + saline	14	25	*P* = 0.652 > 0.05
Levi et al, 2007 [27]	23.8 ± 7.6	24.0 ± 7.4	*P* = 0.90 > 0.05	UFH 5000 units bid (subcutaneously)	Usual care + saline	29.3	31.9	*P* > 0.05
Liu et al, 2014 [28]	20.82 ± 6.5	21.0 ± 6.69	*P* = 0.935 > 0.05	UFH 70 U/kg/24 h 5-7d (vein)	Usual care + saline	31.8	40	*P* = 0.434 > 0.05
Liu et al, 2009 [29]	12.67 ± 4.27	15.73 ± 7.27	*P* = 0.708 > 0.05	UFH 70 U/kg/24 h 5-7d (vein)	Usual care + saline	33.3	36.4	*P* = 0.643 > 0.05
Peng et al, 2013 [30]	15.22 ± 5.34	18.46 ± 5.53	*P* > 0.05	UFH 70 U/kg/24 h 5-7d (vein)	Usual care + saline	12.5	28.6	*P* < 0.05
Wang et al, 2012 [31]	18.7 ± 6.9	15.2 ± 6.2	*P* > 0.05	UFH 3-4 U/kg/h 7d (vein)	Usual care	8.33	12.5	*P* > 0.05
Wu et al*,* 2011 [32]	16.69 ± 4.12	18.89 ± 5.23	*P* = 0.684 > 0.05	UFH 5 U/kg/h 7d (vein)	Usual care	17.78	37.5	*P* < 0.05
Zhang et al, 2006 [33]	NR	NR	*P* > 0.05	UFH 3-4 U/kg/h 7d (vein)	Usual care	27.3	27.3	*P* < 0.05
Zhao et al, 2009 [34]	14.5 ± 4.2	14.8 ± 6.2	*P* > 0.05	UFH 40-50 mg/kg/24 h 5-7d (vein)	Usual care	15.4	32.4	*P* = 0.03 < 0.05
Zhao et al, 2007 [35]	20.54 ± 4.8	18.19 ± 3.69	*P* > 0.05	UFH 300 U/h 5d (vein)	Usual care + saline	33.3	48	*P* > 0.05
Zhang et al*,* 2016 [36]	11.7 ± 3.1	13.7 ± 7.6	*P* > 0.05	UFH 5 U/kg/h 7d (vein)	Usual care	NR	NR	NR

*NR* not reported, *UFH* unfractionated heparin, *APACHE II* Acute Physiology and Chronic Health Evaluation II, *IQR* interquartile range

Moreover, after calculation, the result showed that the median value of APACHE II score could be used to estimate its mean in Jaimes et al’ trial. In Supplementary Fig. S1, the X-axis was RR value and the Y-axis was SE (log [RR]). The outer dashed lines indicate the triangular region within which 95% of studies are expected to lie in the absence of biases and heterogeneity. The solid vertical line corresponds to no intervention effect. The funnel plot was basically symmetrical, indicating a small risk of publication bias. As shown in Table 2, there were various doses of UFH administration in different researches. The route of UFH administration was intravenous for 1 week in most researches. Our group usually use 100 U/kg UFH by continuous intravenous pumping in clinic work. Furthermore, a multicenter RCT (NCT02654561) on the effect of UFH in sepsis is ongoing by our team [37].

### Quality assessment

**Fig. 2:**
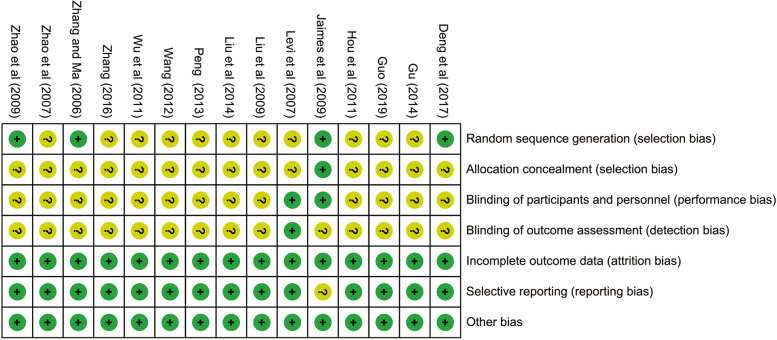
Risk of bias summary. Review author’s judgements about each included study. Low, moderate and high risk of bias are represented as green, yellow, and red colors respectively

**Fig. 3:**
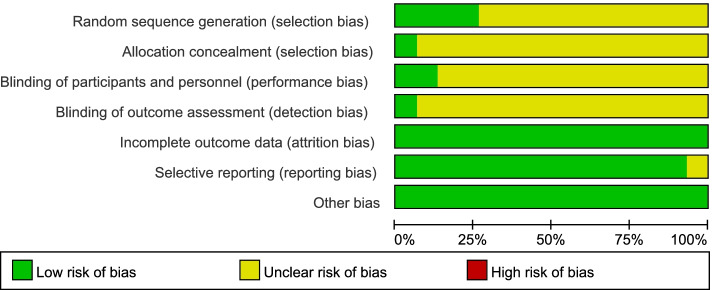
Risk of bias graph. Review author’ judgements about each risk item presented as percentages across all included studies

Main reasons for bias for individual studies were shown in Fig. 2 and Fig. 3, each risk of bias item was presented as percentages. In general, all included studies were usually classified as moderate quality. Random sequence generation, allocation concealment, blinding of outcome assessments, participants and personnel were unclear or seldom reported in these trials. But adequate outcome data was reported in all studies and selective reporting was low for most studies. The strength of evidence assessment was shown in Supplementary Table S3. It showed that the 28 d mortality and PLT were high quality as outcome indicators.

### Primary outcome

**Fig. 4:**
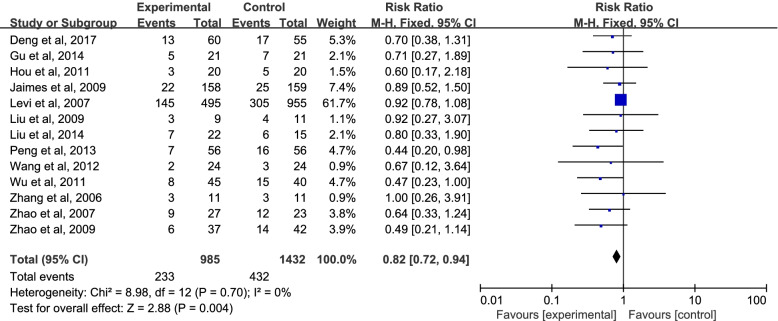
Mortality in patients randomized to UFH versus placebo or usual care. CI, confidence interval; RR, risk ratio

The forest plot for 28 d mortality was shown in Fig. 4. Within 28 days of admission, 236 (23.9%) died in the UFH group, and 429 (30%) died in the control group (RR = 0.84; 95% CI = 0.73 to 0.96; *P* = 0.009 < 0.05), indicating a statistically significant reduction in 28 d mortality in UFH-treated patients with sepsis. There was no evidence of heterogeneity between studies (I^2^ = 0.0%).

### Subgroup analysis

**Fig. 5:**
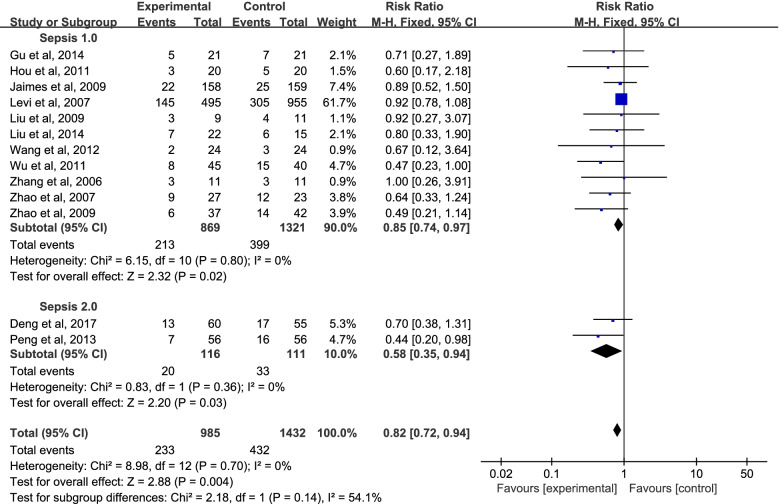
Subgroup analysis of mortality. CI, confidence interval; RR, risk ratio

Subgroup analysis was utilized to identify the effect of UFH on sepsis which is based on the various definitions and diagnostic criteria of sepsis. As Fig. 5 showed, except the Deng’s and Peng’s studies [22, 30], all the other studies were included in the group of Sepsis 1.0, with the RR = 0.85; 95% CI = 0.74 to 0.97; *P* = 0.02 < 0.05; I^2^ = 0.0%. And for Sepsis 2.0, with the RR = 0.58; 95% CI = 0.35 to 0.94; *P* = 0.03 < 0.05; I^2^ =0.0%. The 28 d mortality of patients treated with UFH was statistically significant in both sepsis 1.0 group and sepsis 2.0 group. There was no evidence of heterogeneity between studies (I^2^ = 0.0%). There was high heterogeneity among subgroup studies (I^2^ = 54.1%). The finding from this subgroup analysis further reflects the benefit of UFH for treating septic patients. In clinical practice, APACHE II score was widely used to indicate the disease severity and predict the prognosis of critically ill patients. To date, there is no unified international rules relating to APACHE II cut-off value. In this trial, the mean of APACHE II score ranged from 9 to 23.8 for patients in UFH group, 13, 15, 17 and 19 were selected as APACHE II cut-off values respectively. On the basis of various APACHE II scores, subgroup analysis was performed (Table 3).

**Table 3:** The pooled RR values under various APACHE II scores

APACHE II cut-off	Studies	Participants	RR (95% CI)	*P*
APACHE II ≤ 13	3	399	0.81 [0.53, 1.26]	0.36
APACHE II > 13	9	1996	0.82 [0.71, 0.94]	0.006
APACHE II ≤ 15	5	498	0.74 [0.51, 1.07]	0.11
APACHE II > 15	7	1897	0.83 [0.72, 0.96]	0.01
APACHE II ≤ 17	7	695	0.63 [0.47, 0.86]	0.003
APACHE II > 17	5	1700	0.88 [0.76, 1.03]	0.10
APACHE II ≤ 19	8	743	0.64 [0.47, 0.86]	0.003
APACHE II > 19	4	1652	0.88 [0.76, 1.03]	0.11
APACHE II ≤ 13	4	419	0.82 [0.55, 1.24]	0.36
13 < APACHE II ≤ 17	3	276	0.46 [0.29, 0.74]	0.001
APACHEII > 17	5	1700	0.88 [0.76, 1.03]	0.10
Total	12	2395	0.82 [0.71, 0.94]	0.004

*APACHE II* Acute Physiology and Chronic Health Evaluation II, *RR* Relative risk

Among 13 trials, except Zhang et al’ study [33], the other 12 studies were included in the subgroup analysis. The patients with APACHE II scores between 13 and 17 might benefit from UFH treatment. Subsequently, APACHE II score of 15 was selected as the ultimate cut-off value which could provide the best diagnostic accuracy to predict mortality of critical ill patients [38].

**Fig. 6:**
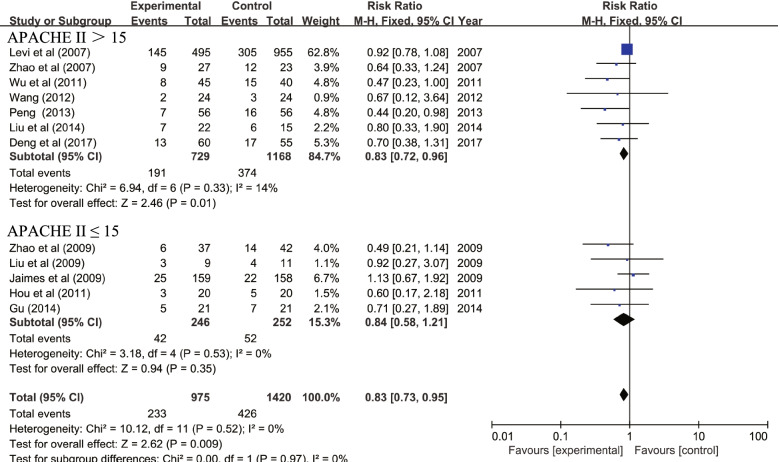
Subgroup analysis of mortality in which APACHE II cut-off value is 15. APACHE II, Acute Physiology and Chronic Health of Evaluation II; CI, confidence; RR, risk ratio

As Fig. 6 showed, five studies [23, 25, 26, 29, 34] were comprised in the group of APACHE II ≤ 15, with the RR = 0.84; 95% CI = 0.58 to 1.21; *P* = 0.35 > 0.05; I^2^ = 0.0%. And seven studies [22, 27, 28, 30–32, 35] were comprised in the group of APACHE II > 15, with the RR = 0.83; 95% CI = 0.72 to 0.96; *P* = 0.01 < 0.05; I^2^ = 14.0%. Because the I^2^ < 30%, the heterogeneity could be ignored. UFH treatment might be beneficial in patients with APACHE II > 15 but not with APACHE II ≤ 15.

### Coagulation indicators

There were eight studies [22, 25, 28, 32–36] reported PLT, PT and APTT, respectively. In total, 277 participants were enrolled in the UFH group and 262 participants were enrolled in the control group. As shown in the forest plot (Supplementary Fig. S2, S3 and S4), the *P* values were 0.03, 0.93 and 0.007, respectively. The PLT (MD = 9.18; 95% CI = 0.68 to 17.68; *P* = 0.03 < 0.05; I^2^ = 21%) in UFH group was higher than that in control group. The PT results (MD = − 0.05; 95% CI = − 1.34 to 1.23; *P* = 0.93 > 0.05; I^2^ = 81%) failed to reach statistical significance, although the considerable heterogeneity, the APTT in UFH group was shorter than that in control group (MD = − 8.01 95% CI = − 13.84 to − 2.18; *P* = 0.007 < 0.05; I^2^ = 94%).

### Secondary outcomes

UFH reduced the incidence of MODS and the LOS in ICU. Four studies [22, 28, 30, 34] compared the incidence of MODS as Supplementary Fig. S5 shown. In summary, 177 participants were enrolled in the UFH group and 169 participants were enrolled in the control group. Comparing to control group, UFH reduced MODS incidence with statistical significance (RR = 0.61 95% CI = 0.45 to 0.84, *P* = 0.002 < 0.05; I^2^ = 0%).

Five researches [24, 25, 28, 30, 33] evaluated the effect of UFH on LOS in ICU in sepsis patients. Forest plot (Supplementary Fig. S6) showed the results. In total, 154 participants were enrolled in the UFH group and 147 participants were enrolled in the control group. Comparing to control group, UFH reduced LOS in ICU with statistical significance (MD = − 4.94 95% CI = − 6.89 to − 2.99, *P* < 0.00001 < 0.05; I^2^ = 66%). But the heterogeneity was considerable.

Four studies [24, 28, 30, 34] reported the duration of ventilation (Supplementary Fig. S7). Generally, 160 participants were enrolled in the UFH group and 158 participants were enrolled in the control group. Comparing to control group, UFH decreased ventilation time with statistical significance (MD = − 3.01 95%; CI = − 4.00 to − 2.02, *P* < 0.00001 < 0.05; I^2^ = 0%).

### Bleeding complications

Four studies reported the bleeding complications [22, 26, 27, 32] (Supplementary Fig. S8). Seven hundred fifty-one participants were enrolled in the UFH group and 1211 participants were enrolled in the control group totally. There were 15 (1.9%) bleeding events in the UFH group and 15 (1.2%) in the control group (RR = 1.10; 95% CI = 0.54 to 2.23; *P* = 0.80 > 0.05; I^2^ = 0%). There were no statistically significant differences. UFH had no effect on bleeding events in patients with sepsis.

## Discussion

In this study, we demonstrated that UFH was an effective treatment for sepsis. The 28 d mortality was relatively reduced by 16% in the UFH group. What’s more, the 28 d mortality of sepsis patients with APACHE II > 15 was relatively reduced by 17% in the UFH group. There were no significant bleeding complications in UFH group, which indicated the safety of UFH. To our knowledge, our study is the first meta-analysis to focus on the effect of UFH in sepsis.

Almost all the sepsis patients experienced coagulation abnormalities, ranging from minor changes that could only be detected by extremely sensitive tests to DIC [8]. Many anticoagulants have been examined to ameliorate the mortality in recent years, such as the tissue factor pathway inhibitor (TFPI), APC and thrombomodulin (TM) [39–41]. However, none of them has been proven to be effective in sepsis. Recently, a SCARLET RCT showed that administration of a recombinant human thrombomodulin (rhTM) did not significantly reduce 28 d all-cause mortality among patients with sepsis-induced coagulopathy compared with placebo [42]. The reasons for the negative results may be multifactorial, including long study period, large differences in the number of enrolled patients from different ICUs, concurrent using of heparin, long time interval from diagnosis to the first dose of rhTM and selected end-point. Therefore, this does not mean that anticoagulant therapy is ineffective. The results of this research ought to be interpreted critically.

The role of UFH in sepsis is much more than just an anticoagulant [43]. Our team have identified the beneficial effects of UFH clinically [33, 34]. The crucial role of UFH in sepsis was further demonstrated in vivo and in vitro. UFH prevented lethality in endotoxemic mice [44]. UFH interfered with Krüppel-like factor 5 (KLF-5) mediated nuclear factor-кB (NF-kB) activation and contributed to the inhibitory effects of chemokines and monocytes migration [45]. Concurrently, UFH enhanced endothelial barrier function and angiopoietin (Ang)/Tie2 axis probably represented one of the mechanisms by which UFH exerted its protective effect [46]. A therapeutic dose of UFH could also protect glycocalyx from shedding by inhibiting inflammation [47].

The conclusion of this meta-analysis was consistent with previous reports. Multiple lines of evidence suggested that UFH may improve clinical outcomes in sepsis. UFH is more suitable in China as well because it is widely available and inexpensive. Higher quality evidence is needed to guide clinical practice [48].

To date, there were three meta-analyses on the role of heparin in sepsis published in English. Wang et al’s meta-analysis published in 2014 concluded that heparin therapy reduced 28 d mortality in adult severe sepsis patients [13]. There were four main differences between the design of two studies. 1. Both the RCTs and NRCTs were taken into consideration in Wang et al’s research. We only contained the RCTs. 2. Wang et al’s research included trials on both UFH and LMWH as the intervention. We only analyzed the effects of UFH. 3.The patients who received continuous renal replacement therapy (CRRT) were excluded in our research since the use of anticoagulants during CRRT might affect the results. Wang et al’s research did not exclude such patients. 4. We included studies from 2006 to 2021.

Subsequently, Zarychanski et al’s meta-analysis was published in 2015, which showed that heparin was associated with decreased mortality in patients with sepsis, septic shock and DIC [14]. The distinction of two studies contained the following two aspects: first, Zarychanski et al’s research involved trials relating to LMWH and other anticoagulants, but UFH was the only intervention in our research. Second, we drew the conclusion from literature within 15 years. Zarychanski et al’ research involved literature from 1983 to 2014, so the heterogeneity was obvious.

The third meta-analysis was about the efficacy and safety of LMWH in patients with sepsis [15]. There are three differences from our research. First, in contrast with Yu et al’s team, both English and non-English researches were included in our study which could decrease potential publication bias, while only Chinese studies were analyzed in Fan et al. ‘s research. Second, the larger sample the clinical trial contained, the more representative the research’s conclusion was. The number of patients enrolled in our research was almost four times than that in Fan et al’s research. Third, Fan et al’s research evaluated the effects of LMWH in sepsis, whereas we studied the role of UFH. The action mechanisms of the two heparins are different, UFH seems to be more promising.

There are several advantages in this article. First, we enrolled studies both in English and in Chinese in this research, which meant the generalization of the current findings. Second, to our knowledge, our study is the first meta-analysis to focus on the effect of UFH in sepsis. Last, the large number of subjects included and the diversity in patients’ characteristics provided diverse information.

The study also has several limitations. First, as shown in the forest plot, the participants in Levi et al’s study took up more than half of the total number and all of them received recombinant activated protein C, which has been withdrawal from the market. Second, due to the various publication years, the definitions of sepsis, septic shock and DIC had changed. In 2016, the Sepsis-3 Task Force updated the clinical criteria for sepsis, excluding the need for systemic inflammatory response syndrome (SIRS) and the concept of severe sepsis [7]. Third, bleeding and other adverse events were incompletely reported which may influence the accuracy of results. For example, there were only four of fifteen trials reported bleeding information. Fourth, the doses of UFH and the treatment duration varied. Last, overall quality of the body of evidence was not high enough and there was a lack of multicenter RCTs.

## Conclusion

UFH may reduce 28 d mortality and improve the clinical efficacy for sepsis patients without bleeding complications. We call for large multicenter RCTs to evaluate the clinical value of UFH in sepsis patients because of the moderate quality of this body of evidence.

## Supplementary Information

**Additional file 1 **: **Figure S1**. The funnel plot of 28 d mortality.

**Additional file 2 **: **Figure S2**. The forest plot of PLT.

**Additional file 3 **: **Figure S3**. The forest plot of PT.

**Additional file 4 **: **Figure S4**. The forest plot of APTT.

**Additional file 5 **: **Figure S5**. The forest plot of MODS incidence.

**Additional file 6 Figure S6**. The forest plot of LOS in ICU.

**Additional file 7 **: **Figure S7**. The forest plot of the duration of Ventilation.

**Additional file 8 **: **Figure S8**. The forest plot of bleeding complication.

**Additional file 9 **: **Table S1**. The PubMed strategy.

**Additional file 10 **: **Table S2**. Excluded literature information.

**Additional file 11 **: **Table S3**. Evidence assessment.

## Data Availability

Not applicable.

## Abbreviations

Ang: Angiopoietin
APACHE II: Acute Physiology and Chronic Health Evaluation II
APC: Activated protein C
APTT: Activated partial thromboplastin time
AT: Antithrombin
CIs: Confidence intervals
CMeSH: Chinese Medical Subject Headings
CRRT: Continuous Renal replacement therapy
DIC: Disseminated intravascular coagulation
F: Factor
IQR: Interquartile range
ISTH: International Society on Thrombosis & Haemostasis
KLF-5: Krüppel-like factor 5
LMWH: Low molecular weight heparin
LOS: Length of stay
MeSH: Medical Subject Headings
MD: Mean differences
MODS: Multiple organ dysfunction syndrome
NF-kB: Nuclear factor-кB
PLT: Platelet
PRISMA: Preferred Reporting Items for the Systematic Reviews and Meta-Analyses
PT: Prothrombin time
RCTs: Randomized controlled trials
RevMan: Review Manager
rhAPC: Recombinant human APC
rhTM: Recombinant human thrombomodulin
RoB2: Risk of Bias tool 2
RR: Relative risk
SIRS: Systemic inflammatory response syndrome
SSC: Surviving Sepsis Campaign
TFPI: Tissue factor pathway inhibitor
TM: Thrombomodulin
UFH: Unfractionated heparin
