# Supplementary figures and images for: Unfractionated heparin improves the clinical efficacy in adult sepsis patients: a systematic review and meta-analysis
Source: BMC Anesthesiol. 2022 Jan 21;22:28. doi: 10.1186/s12871-021-01545-w (PMC8777179; doi:10.1186/s12871-021-01545-w)

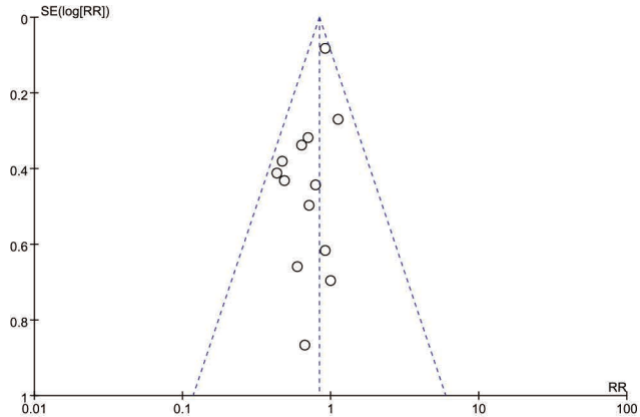

Supplement: Supplementary file 1 — Additional file 1 : Figure S1. The funnel plot of 28 d mortality. [file 12871_2021_1545_MOESM1_ESM.pdf]

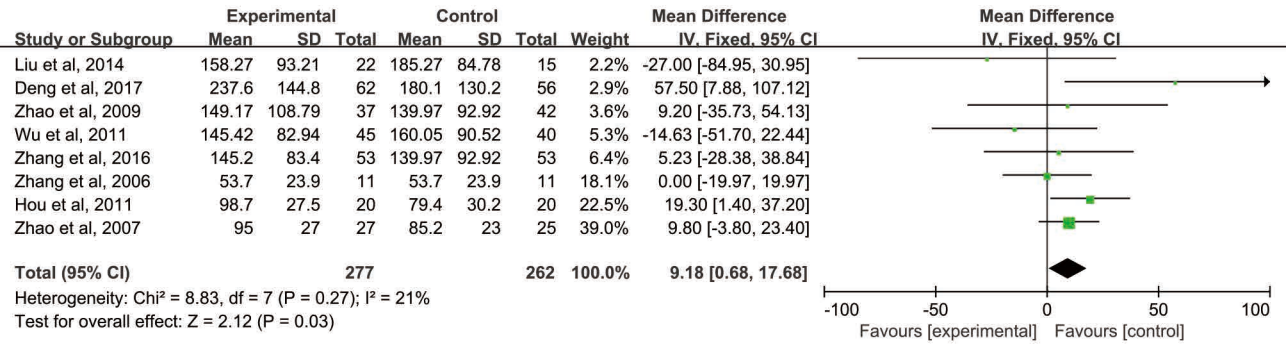

Supplement: Supplementary file 2 — Additional file 2 : Figure S2. The forest plot of PLT. [file 12871_2021_1545_MOESM2_ESM.pdf]

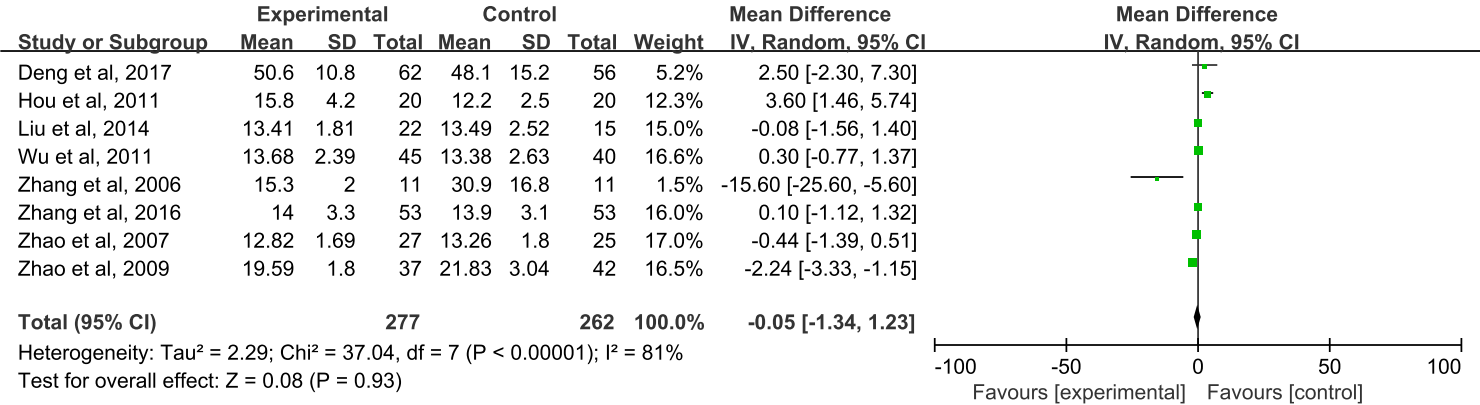

Supplement: Supplementary file 3 — Additional file 3 : Figure S3. The forest plot of PT. [file 12871_2021_1545_MOESM3_ESM.pdf]

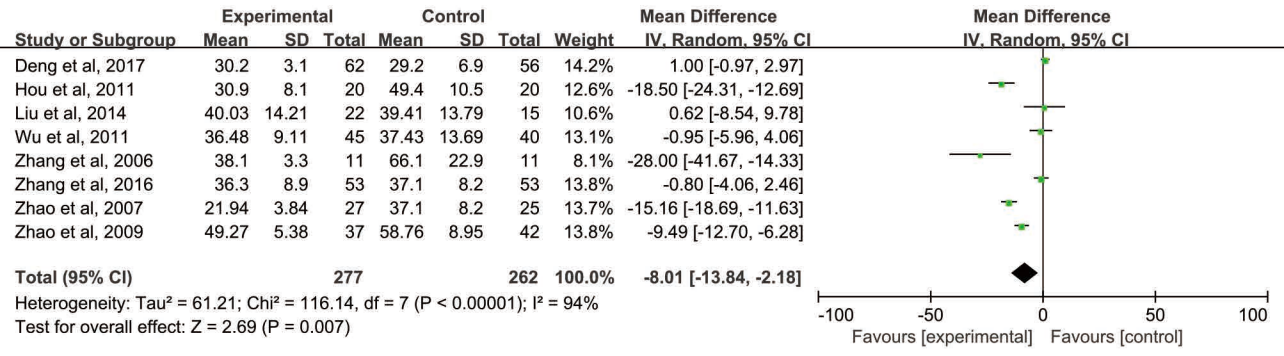

Supplement: Supplementary file 4 — Additional file 4 : Figure S4. The forest plot of APTT. [file 12871_2021_1545_MOESM4_ESM.pdf]

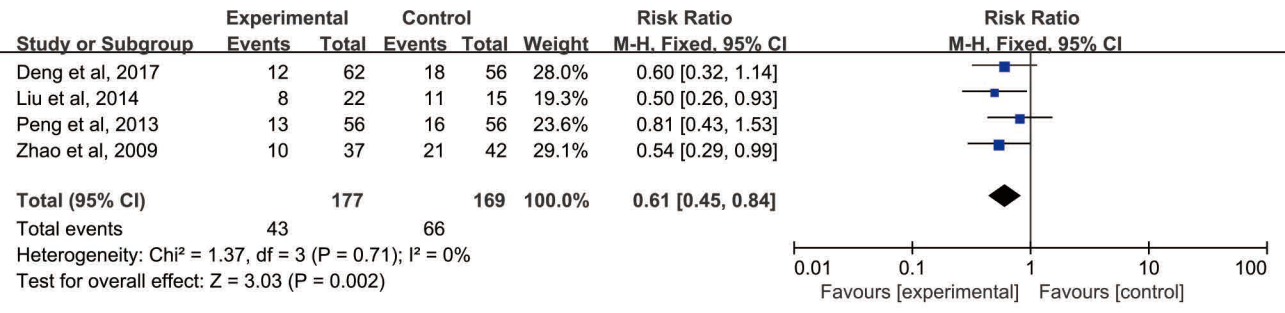

Supplement: Supplementary file 5 — Additional file 5 : Figure S5. The forest plot of MODS incidence. [file 12871_2021_1545_MOESM5_ESM.pdf]

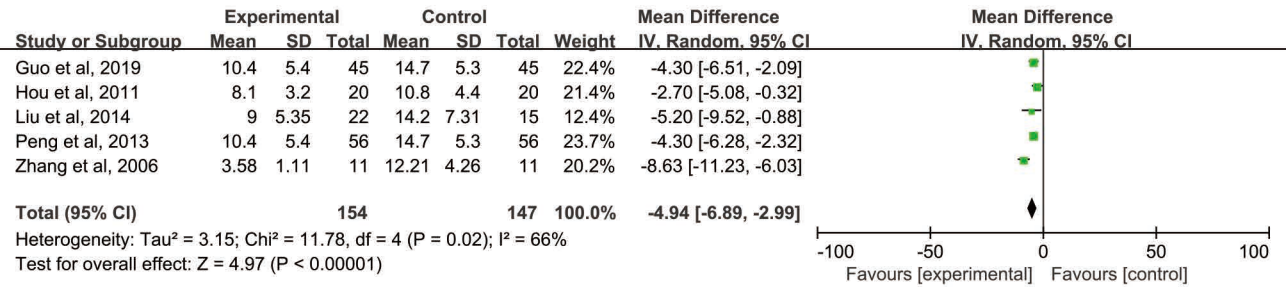

Supplement: Supplementary file 6 — Additional file 6 Figure S6. The forest plot of LOS in ICU. [file 12871_2021_1545_MOESM6_ESM.pdf]

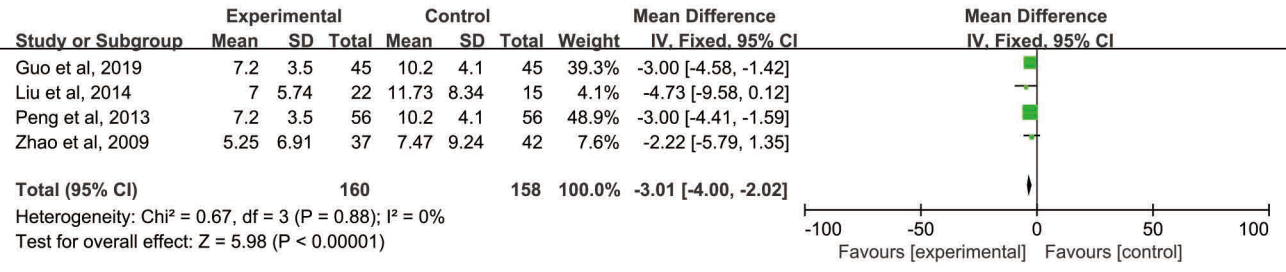

Supplement: Supplementary file 7 — Additional file 7 : Figure S7. The forest plot of the duration of Ventilation. [file 12871_2021_1545_MOESM7_ESM.pdf]

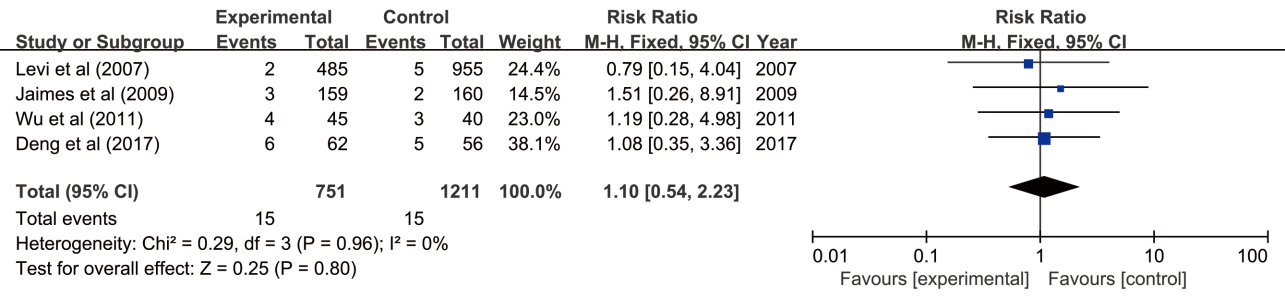

Supplement: Supplementary file 8 — Additional file 8 : Figure S8. The forest plot of bleeding complication. [file 12871_2021_1545_MOESM8_ESM.pdf]
