# Supplementary material for: Unfractionated heparin improves the clinical efficacy in adult sepsis patients: a systematic review and meta-analysis
Source: BMC Anesthesiol. 2022 Jan 21;22:28. doi: 10.1186/s12871-021-01545-w (PMC8777179; doi:10.1186/s12871-021-01545-w)
Supplement: Supplementary file 9 — Additional file 9 : Table S1. The PubMed strategy. [file 12871_2021_1545_MOESM9_ESM.docx]

| #1 | sepsis [MeSH Terms],,,"""sepsis""[MeSH Terms]","121,702" |
| --- | --- |
| #2 | "sepsis or Pyemia or Pyemias or Pyohemia or Pyohemias or Pyaemia or Pyaemias or Septicemia or Septicemias or ""Poisoning, Blood"" or ""Blood Poisoning"" or ""Blood Poisonings"" or ""Poisonings, Blood"" or ""Severe Sepsis"" or ""Sepsis, Severe"" or ""septic shock"" or "" shock, septic"" or bactermia or "" systemic inflammatory response syndrome""",,,"((((((((((((((((((""sepsis""[MeSH Terms] OR ""sepsis""[All Fields]) OR (((""sepsis""[MeSH Terms] OR ""sepsis""[All Fields]) OR ""pyaemia""[All Fields]) OR ""pyemia""[All Fields])) OR ((""sepsis""[MeSH Terms] OR ""sepsis""[All Fields]) OR ""pyemias""[All Fields])) OR ((""sepsis""[MeSH Terms] OR ""sepsis""[All Fields]) OR ""pyohemia""[All Fields])) OR ((""sepsis""[MeSH Terms] OR ""sepsis""[All Fields]) )) OR (((""sepsis""[MeSH Terms] OR ""sepsis""[All Fields]) OR ""pyaemia""[All Fields]) OR ""pyemia""[All Fields])) OR ((""sepsis""[MeSH Terms] OR ""sepsis""[All Fields]) )) OR (((((""sepsis""[MeSH Terms] OR ""sepsis""[All Fields]) OR ""septicaemias""[All Fields]) OR ""septicemias""[All Fields]) OR ""septicaemia""[All Fields]) OR ""septicemia""[All Fields])) OR (((((""sepsis""[MeSH Terms] OR ""sepsis""[All Fields]) OR ""septicaemias""[All Fields]) OR ""septicemias""[All Fields]) OR ""septicaemia""[All Fields]) OR ""septicemia""[All Fields])) OR ""poisoning blood""[All Fields]) OR ""Blood Poisoning""[All Fields]) OR ""Blood Poisonings""[All Fields]) OR (((""sepsis""[MeSH Terms] OR ""sepsis""[All Fields]) OR (""poisonings""[All Fields] AND ""blood""[All Fields])) )) OR ""Severe Sepsis""[All Fields]) OR ""sepsis severe""[All Fields]) OR ""septic shock""[All Fields]) OR ""shock septic""[All Fields]) OR ""bactermia""[All Fields]) OR ""systemic inflammatory response syndrome""[All Fields]","204,304" |
| #3 | "(sepsis[MeSH Terms]) OR (sepsis or Pyemia or Pyemias or Pyohemia or Pyohemias or Pyaemia or Pyaemias or Septicemia or Septicemias or ""Poisoning, Blood"" or ""Blood Poisoning"" or ""Blood Poisonings"" or ""Poisonings, Blood"" or ""Severe Sepsis"" or ""Sepsis, Severe"" or ""septic shock"" or "" shock, septic"" or bactermia or "" systemic inflammatory response syndrome"")",,,"""sepsis""[MeSH Terms] OR (((((((((((((((((((""sepsis""[MeSH Terms] OR ""sepsis""[All Fields]) OR (((""sepsis""[MeSH Terms] OR ""sepsis""[All Fields]) OR ""pyaemia""[All Fields]) OR ""pyemia""[All Fields])) OR ((""sepsis""[MeSH Terms] OR ""sepsis""[All Fields]) OR ""pyemias""[All Fields])) OR ((""sepsis""[MeSH Terms] OR ""sepsis""[All Fields]) OR ""pyohemia""[All Fields])) OR ((""sepsis""[MeSH Terms] OR ""sepsis""[All Fields]) )) OR (((""sepsis""[MeSH Terms] OR ""sepsis""[All Fields]) OR ""pyaemia""[All Fields]) OR ""pyemia""[All Fields])) OR ((""sepsis""[MeSH Terms] OR ""sepsis""[All Fields]) )) OR (((((""sepsis""[MeSH Terms] OR ""sepsis""[All Fields]) OR ""septicaemias""[All Fields]) OR ""septicemias""[All Fields]) OR ""septicaemia""[All Fields]) OR ""septicemia""[All Fields])) OR (((((""sepsis""[MeSH Terms] OR ""sepsis""[All Fields]) OR ""septicaemias""[All Fields]) OR ""septicemias""[All Fields]) OR ""septicaemia""[All Fields]) OR ""septicemia""[All Fields])) OR ""poisoning blood""[All Fields]) OR ""Blood Poisoning""[All Fields]) OR ""Blood Poisonings""[All Fields]) OR (((""sepsis""[MeSH Terms] OR ""sepsis""[All Fields]) OR (""poisonings""[All Fields] AND ""blood""[All Fields])) )) OR ""Severe Sepsis""[All Fields]) OR ""sepsis severe""[All Fields]) OR ""septic shock""[All Fields]) OR ""shock septic""[All Fields]) OR ""bactermia""[All Fields]) OR ""systemic inflammatory response syndrome""[All Fields])","204,304" |
| #4 | """Unfractionated Heparin"" or ""Heparin, Unfractionated"" or ""Heparinic Acid"" or Liquaemin or ""Sodium Heparin"" or ""Heparin, Sodium"" or ""Heparin Sodium"" or alpha-Heparin or ""alpha Heparin"" or heparin",,,"((((((((""Unfractionated Heparin""[All Fields] OR ""heparin unfractionated""[All Fields]) OR ""Heparinic Acid""[All Fields]) OR ((""heparin""[MeSH Terms] OR ""heparin""[All Fields]) OR ""liquaemin""[All Fields])) OR ""Sodium Heparin""[All Fields]) OR ""Heparin Sodium""[All Fields]) OR ""Heparin Sodium""[All Fields]) OR (((""heparin""[MeSH Terms] OR ""heparin""[All Fields]) OR (""alpha""[All Fields] AND ""heparin""[All Fields])) OR ""alpha Heparin""[All Fields])) OR ""alpha Heparin""[All Fields]) OR (((((((((((((((""heparin""[MeSH Terms] OR ""heparin""[All Fields]) OR ""heparine""[All Fields]) OR ""heparins""[All Fields]) OR ""heparin s""[All Fields]) OR ""heparinate""[All Fields]) OR ""heparinated""[All Fields]) OR ""heparines""[All Fields]) OR ""heparinic""[All Fields]) OR ""heparinisation""[All Fields]) OR ""heparinised""[All Fields]) ) OR ""heparinization""[All Fields]) OR ""heparinize""[All Fields]) OR ""heparinized""[All Fields]) OR ""heparinizing""[All Fields])","103,957" |
| #5 | heparin [MeSH Terms],,,"""heparin""[MeSH Terms]","64,117" |
| #6 | "(heparin[MeSH Terms]) OR (""Unfractionated Heparin"" or ""Heparin, Unfractionated"" or ""Heparinic Acid"" or Liquaemin or ""Sodium Heparin"" or ""Heparin, Sodium"" or ""Heparin Sodium"" or alpha-Heparin or ""alpha Heparin"" or heparin)",,,"""heparin""[MeSH Terms] OR (((((((((""Unfractionated Heparin""[All Fields] OR ""heparin unfractionated""[All Fields]) OR ""Heparinic Acid""[All Fields]) OR ((""heparin""[MeSH Terms] OR ""heparin""[All Fields]) OR ""liquaemin""[All Fields])) OR ""Sodium Heparin""[All Fields]) OR ""Heparin Sodium""[All Fields]) OR ""Heparin Sodium""[All Fields]) OR (((""heparin""[MeSH Terms] OR ""heparin""[All Fields]) OR (""alpha""[All Fields] AND ""heparin""[All Fields])) OR ""alpha Heparin""[All Fields])) OR ""alpha Heparin""[All Fields]) OR (((((((((((((((""heparin""[MeSH Terms] OR ""heparin""[All Fields]) OR ""heparine""[All Fields]) OR ""heparins""[All Fields]) OR ""heparin s""[All Fields]) OR ""heparinate""[All Fields]) OR ""heparinated""[All Fields]) OR ""heparines""[All Fields]) OR ""heparinic""[All Fields]) OR ""heparinisation""[All Fields]) OR ""heparinised""[All Fields]) ) OR ""heparinization""[All Fields]) OR ""heparinize""[All Fields]) OR ""heparinized""[All Fields]) OR ""heparinizing""[All Fields]))","103,957" |
| #7 | """Disseminated Coagulation, Intravascular"" or ""Coagulation, Intravascular Disseminated"" or ""Coagulations, Intravascular Disseminated"" or ""Disseminated Coagulations, Intravascular"" or ""Intravascular Disseminated Coagulations"" or ""Intravascular Coagulation, Disseminated"" or ""Intravascular Disseminated Coagulation"" or ""Coagulation, Disseminated Intravascular"" or ""Coagulations, Disseminated Intravascular"" or ""Disseminated Intravascular Coagulations"" or ""Intravascular Coagulations, Disseminated"" or ""Consumption Coagulopathy"" or ""Coagulopathies, Consumption"" or ""Coagulopathy, Consumption"" or ""Consumption Coagulopathies""",,,"(((((((((((((((((""disseminated intravascular coagulation""[MeSH Terms] OR ((""disseminated""[All Fields] AND ""intravascular""[All Fields]) AND ""coagulation""[All Fields])) OR ""disseminated intravascular coagulation""[All Fields]) OR ((""disseminated""[All Fields] AND ""coagulation""[All Fields]) AND ""intravascular""[All Fields])) ) OR ((((""disseminated intravascular coagulation""[MeSH Terms] OR ((""disseminated""[All Fields] AND ""intravascular""[All Fields]) AND ""coagulation""[All Fields])) OR ""disseminated intravascular coagulation""[All Fields]) OR ((""coagulation""[All Fields] AND ""intravascular""[All Fields]) AND ""disseminated""[All Fields])) )) OR ((((""disseminated intravascular coagulation""[MeSH Terms] OR ((""disseminated""[All Fields] AND ""intravascular""[All Fields]) AND ""coagulation""[All Fields])) OR ""disseminated intravascular coagulation""[All Fields]) OR ((""coagulations""[All Fields] AND ""intravascular""[All Fields]) AND ""disseminated""[All Fields])) )) OR ((((""disseminated intravascular coagulation""[MeSH Terms] OR ((""disseminated""[All Fields] AND ""intravascular""[All Fields]) AND ""coagulation""[All Fields])) OR ""disseminated intravascular coagulation""[All Fields]) OR ((""disseminated""[All Fields] AND ""coagulations""[All Fields]) AND ""intravascular""[All Fields])) )) OR ((((""disseminated intravascular coagulation""[MeSH Terms] OR ((""disseminated""[All Fields] AND ""intravascular""[All Fields]) AND ""coagulation""[All Fields])) OR ""disseminated intravascular coagulation""[All Fields]) OR ((""intravascular""[All Fields] AND ""disseminated""[All Fields]) AND ""coagulations""[All Fields])) )) OR ""intravascular coagulation disseminated""[All Fields]) OR ""Intravascular Disseminated Coagulation""[All Fields]) OR ""coagulation disseminated intravascular""[All Fields]) OR ((((""disseminated intravascular coagulation""[MeSH Terms] OR ((""disseminated""[All Fields] AND ""intravascular""[All Fields]) AND ""coagulation""[All Fields])) OR ""disseminated intravascular coagulation""[All Fields]) OR ((""coagulations""[All Fields] AND ""disseminated""[All Fields]) AND ""intravascular""[All Fields])) )) OR ""Disseminated Intravascular Coagulations""[All Fields]) OR ((((""disseminated intravascular coagulation""[MeSH Terms] OR ((""disseminated""[All Fields] AND ""intravascular""[All Fields]) AND ""coagulation""[All Fields])) OR ""disseminated intravascular coagulation""[All Fields]) OR ((""intravascular""[All Fields] AND ""coagulations""[All Fields]) AND ""disseminated""[All Fields])) )) OR ""Consumption Coagulopathy""[All Fields]) OR ((((""disseminated intravascular coagulation""[MeSH Terms] OR ((""disseminated""[All Fields] AND ""intravascular""[All Fields]) AND ""coagulation""[All Fields])) OR ""disseminated intravascular coagulation""[All Fields]) OR (""coagulopathies""[All Fields] AND ""consumption""[All Fields])) )) OR ""coagulopathy consumption""[All Fields]) OR ""Consumption Coagulopathies""[All Fields]","16,159" |
| #8 | """Disseminated Intravascular Coagulation"" [MeSH Terms]",,,"""Disseminated Intravascular Coagulation""[MeSH Terms]","10,946" |
| #9 | "(""Disseminated Coagulation, Intravascular"" or ""Coagulation, Intravascular Disseminated"" or ""Coagulations, Intravascular Disseminated"" or ""Disseminated Coagulations, Intravascular"" or ""Intravascular Disseminated Coagulations"" or ""Intravascular Coagulation, Disseminated"" or ""Intravascular Disseminated Coagulation"" or ""Coagulation, Disseminated Intravascular"" or ""Coagulations, Disseminated Intravascular"" or ""Disseminated Intravascular Coagulations"" or ""Intravascular Coagulations, Disseminated"" or ""Consumption Coagulopathy"" or ""Coagulopathies, Consumption"" or ""Coagulopathy, Consumption"" or ""Consumption Coagulopathies"") OR (""Disseminated Intravascular Coagulation"" [MeSH Terms])",,,"((((((((((((((((((""Disseminated Intravascular Coagulation""[MeSH Terms] OR ((""disseminated""[All Fields] AND ""intravascular""[All Fields]) AND ""coagulation""[All Fields])) OR ""Disseminated Intravascular Coagulation""[All Fields]) OR ((""disseminated""[All Fields] AND ""coagulation""[All Fields]) AND ""intravascular""[All Fields])) ) OR ((((""Disseminated Intravascular Coagulation""[MeSH Terms] OR ((""disseminated""[All Fields] AND ""intravascular""[All Fields]) AND ""coagulation""[All Fields])) OR ""Disseminated Intravascular Coagulation""[All Fields]) OR ((""coagulation""[All Fields] AND ""intravascular""[All Fields]) AND ""disseminated""[All Fields])) )) OR ((((""Disseminated Intravascular Coagulation""[MeSH Terms] OR ((""disseminated""[All Fields] AND ""intravascular""[All Fields]) AND ""coagulation""[All Fields])) OR ""Disseminated Intravascular Coagulation""[All Fields]) OR ((""coagulations""[All Fields] AND ""intravascular""[All Fields]) AND ""disseminated""[All Fields])) )) OR ((((""Disseminated Intravascular Coagulation""[MeSH Terms] OR ((""disseminated""[All Fields] AND ""intravascular""[All Fields]) AND ""coagulation""[All Fields])) OR ""Disseminated Intravascular Coagulation""[All Fields]) OR ((""disseminated""[All Fields] AND ""coagulations""[All Fields]) AND ""intravascular""[All Fields])) )) OR ((((""Disseminated Intravascular Coagulation""[MeSH Terms] OR ((""disseminated""[All Fields] AND ""intravascular""[All Fields]) AND ""coagulation""[All Fields])) OR ""Disseminated Intravascular Coagulation""[All Fields]) OR ((""intravascular""[All Fields] AND ""disseminated""[All Fields]) AND ""coagulations""[All Fields])) )) OR ""intravascular coagulation disseminated""[All Fields]) OR ""Intravascular Disseminated Coagulation""[All Fields]) OR ""coagulation disseminated intravascular""[All Fields]) OR ((((""Disseminated Intravascular Coagulation""[MeSH Terms] OR ((""disseminated""[All Fields] AND ""intravascular""[All Fields]) AND ""coagulation""[All Fields])) OR ""Disseminated Intravascular Coagulation""[All Fields]) OR ((""coagulations""[All Fields] AND ""disseminated""[All Fields]) AND ""intravascular""[All Fields])) )) OR ""Disseminated Intravascular Coagulations""[All Fields]) OR ((((""Disseminated Intravascular Coagulation""[MeSH Terms] OR ((""disseminated""[All Fields] AND ""intravascular""[All Fields]) AND ""coagulation""[All Fields])) OR ""Disseminated Intravascular Coagulation""[All Fields]) OR ((""intravascular""[All Fields] AND ""coagulations""[All Fields]) AND ""disseminated""[All Fields])) )) OR ""Consumption Coagulopathy""[All Fields]) OR ((((""Disseminated Intravascular Coagulation""[MeSH Terms] OR ((""disseminated""[All Fields] AND ""intravascular""[All Fields]) AND ""coagulation""[All Fields])) OR ""Disseminated Intravascular Coagulation""[All Fields]) OR (""coagulopathies""[All Fields] AND ""consumption""[All Fields])) )) OR ""coagulopathy consumption""[All Fields]) OR ""Consumption Coagulopathies""[All Fields]) OR ""Disseminated Intravascular Coagulation""[MeSH Terms]","16,159" |
| #10 | "((""Disseminated Coagulation, Intravascular"" or ""Coagulation, Intravascular Disseminated"" or ""Coagulations, Intravascular Disseminated"" or ""Disseminated Coagulations, Intravascular"" or ""Intravascular Disseminated Coagulations"" or ""Intravascular Coagulation, Disseminated"" or ""Intravascular Disseminated Coagulation"" or ""Coagulation, Disseminated Intravascular"" or ""Coagulations, Disseminated Intravascular"" or ""Disseminated Intravascular Coagulations"" or ""Intravascular Coagulations, Disseminated"" or ""Consumption Coagulopathy"" or ""Coagulopathies, Consumption"" or ""Coagulopathy, Consumption"" or ""Consumption Coagulopathies"") OR (""Disseminated Intravascular Coagulation"" [MeSH Terms])) OR ((sepsis[MeSH Terms]) OR (sepsis or Pyemia or Pyemias or Pyohemia or Pyohemias or Pyaemia or Pyaemias or Septicemia or Septicemias or ""Poisoning, Blood"" or ""Blood Poisoning"" or ""Blood Poisonings"" or ""Poisonings, Blood"" or ""Severe Sepsis"" or ""Sepsis, Severe"" or ""septic shock"" or "" shock, septic"" or bactermia or "" systemic inflammatory response syndrome""))",,,"(((((((((((((((((((""Disseminated Intravascular Coagulation""[MeSH Terms] OR ((""disseminated""[All Fields] AND ""intravascular""[All Fields]) AND ""coagulation""[All Fields])) OR ""Disseminated Intravascular Coagulation""[All Fields]) OR ((""disseminated""[All Fields] AND ""coagulation""[All Fields]) AND ""intravascular""[All Fields])) ) OR ((((""Disseminated Intravascular Coagulation""[MeSH Terms] OR ((""disseminated""[All Fields] AND ""intravascular""[All Fields]) AND ""coagulation""[All Fields])) OR ""Disseminated Intravascular Coagulation""[All Fields]) OR ((""coagulation""[All Fields] AND ""intravascular""[All Fields]) AND ""disseminated""[All Fields])) )) OR ((((""Disseminated Intravascular Coagulation""[MeSH Terms] OR ((""disseminated""[All Fields] AND ""intravascular""[All Fields]) AND ""coagulation""[All Fields])) OR ""Disseminated Intravascular Coagulation""[All Fields]) OR ((""coagulations""[All Fields] AND ""intravascular""[All Fields]) AND ""disseminated""[All Fields])) )) OR ((((""Disseminated Intravascular Coagulation""[MeSH Terms] OR ((""disseminated""[All Fields] AND ""intravascular""[All Fields]) AND ""coagulation""[All Fields])) OR ""Disseminated Intravascular Coagulation""[All Fields]) OR ((""disseminated""[All Fields] AND ""coagulations""[All Fields]) AND ""intravascular""[All Fields])) )) OR ((((""Disseminated Intravascular Coagulation""[MeSH Terms] OR ((""disseminated""[All Fields] AND ""intravascular""[All Fields]) AND ""coagulation""[All Fields])) OR ""Disseminated Intravascular Coagulation""[All Fields]) OR ((""intravascular""[All Fields] AND ""disseminated""[All Fields]) AND ""coagulations""[All Fields])) )) OR ""intravascular coagulation disseminated""[All Fields]) OR ""Intravascular Disseminated Coagulation""[All Fields]) OR ""coagulation disseminated intravascular""[All Fields]) OR ((((""Disseminated Intravascular Coagulation""[MeSH Terms] OR ((""disseminated""[All Fields] AND ""intravascular""[All Fields]) AND ""coagulation""[All Fields])) OR ""Disseminated Intravascular Coagulation""[All Fields]) OR ((""coagulations""[All Fields] AND ""disseminated""[All Fields]) AND ""intravascular""[All Fields])) )) OR ""Disseminated Intravascular Coagulations""[All Fields]) OR ((((""Disseminated Intravascular Coagulation""[MeSH Terms] OR ((""disseminated""[All Fields] AND ""intravascular""[All Fields]) AND ""coagulation""[All Fields])) OR ""Disseminated Intravascular Coagulation""[All Fields]) OR ((""intravascular""[All Fields] AND ""coagulations""[All Fields]) AND ""disseminated""[All Fields])) )) OR ""Consumption Coagulopathy""[All Fields]) OR ((((""Disseminated Intravascular Coagulation""[MeSH Terms] OR ((""disseminated""[All Fields] AND ""intravascular""[All Fields]) AND ""coagulation""[All Fields])) OR ""Disseminated Intravascular Coagulation""[All Fields]) OR (""coagulopathies""[All Fields] AND ""consumption""[All Fields])) )) OR ""coagulopathy consumption""[All Fields]) OR ""Consumption Coagulopathies""[All Fields]) OR ""Disseminated Intravascular Coagulation""[MeSH Terms]) OR (""sepsis""[MeSH Terms] OR (((((((((((((((((((""sepsis""[MeSH Terms] OR ""sepsis""[All Fields]) OR (((""sepsis""[MeSH Terms] OR ""sepsis""[All Fields]) OR ""pyaemia""[All Fields]) OR ""pyemia""[All Fields])) OR ((""sepsis""[MeSH Terms] OR ""sepsis""[All Fields]) OR ""pyemias""[All Fields])) OR ((""sepsis""[MeSH Terms] OR ""sepsis""[All Fields]) OR ""pyohemia""[All Fields])) OR ((""sepsis""[MeSH Terms] OR ""sepsis""[All Fields]) )) OR (((""sepsis""[MeSH Terms] OR ""sepsis""[All Fields]) OR ""pyaemia""[All Fields]) OR ""pyemia""[All Fields])) OR ((""sepsis""[MeSH Terms] OR ""sepsis""[All Fields]) )) OR (((((""sepsis""[MeSH Terms] OR ""sepsis""[All Fields]) OR ""septicaemias""[All Fields]) OR ""septicemias""[All Fields]) OR ""septicaemia""[All Fields]) OR ""septicemia""[All Fields])) OR (((((""sepsis""[MeSH Terms] OR ""sepsis""[All Fields]) OR ""septicaemias""[All Fields]) OR ""septicemias""[All Fields]) OR ""septicaemia""[All Fields]) OR ""septicemia""[All Fields])) OR ""poisoning blood""[All Fields]) OR ""Blood Poisoning""[All Fields]) OR ""Blood Poisonings""[All Fields]) OR (((""sepsis""[MeSH Terms] OR ""sepsis""[All Fields]) OR (""poisonings""[All Fields] AND ""blood""[All Fields])) )) OR ""Severe Sepsis""[All Fields]) OR ""sepsis severe""[All Fields]) OR ""septic shock""[All Fields]) OR ""shock septic""[All Fields]) OR ""bactermia""[All Fields]) OR ""systemic inflammatory response syndrome""[All Fields]))","217,315" |
| #11 | "(((""Disseminated Coagulation, Intravascular"" or ""Coagulation, Intravascular Disseminated"" or ""Coagulations, Intravascular Disseminated"" or ""Disseminated Coagulations, Intravascular"" or ""Intravascular Disseminated Coagulations"" or ""Intravascular Coagulation, Disseminated"" or ""Intravascular Disseminated Coagulation"" or ""Coagulation, Disseminated Intravascular"" or ""Coagulations, Disseminated Intravascular"" or ""Disseminated Intravascular Coagulations"" or ""Intravascular Coagulations, Disseminated"" or ""Consumption Coagulopathy"" or ""Coagulopathies, Consumption"" or ""Coagulopathy, Consumption"" or ""Consumption Coagulopathies"") OR (""Disseminated Intravascular Coagulation"" [MeSH Terms])) OR ((sepsis[MeSH Terms]) OR (sepsis or Pyemia or Pyemias or Pyohemia or Pyohemias or Pyaemia or Pyaemias or Septicemia or Septicemias or ""Poisoning, Blood"" or ""Blood Poisoning"" or ""Blood Poisonings"" or ""Poisonings, Blood"" or ""Severe Sepsis"" or ""Sepsis, Severe"" or ""septic shock"" or "" shock, septic"" or bactermia or "" systemic inflammatory response syndrome""))) AND ((heparin[MeSH Terms]) OR (""Unfractionated Heparin"" or ""Heparin, Unfractionated"" or ""Heparinic Acid"" or Liquaemin or ""Sodium Heparin"" or ""Heparin, Sodium"" or ""Heparin Sodium"" or alpha-Heparin or ""alpha Heparin"" or heparin))",,,"((((((((((((((((((((""Disseminated Intravascular Coagulation""[MeSH Terms] OR ((""disseminated""[All Fields] AND ""intravascular""[All Fields]) AND ""coagulation""[All Fields])) OR ""Disseminated Intravascular Coagulation""[All Fields]) OR ((""disseminated""[All Fields] AND ""coagulation""[All Fields]) AND ""intravascular""[All Fields])) ) OR ((((""Disseminated Intravascular Coagulation""[MeSH Terms] OR ((""disseminated""[All Fields] AND ""intravascular""[All Fields]) AND ""coagulation""[All Fields])) OR ""Disseminated Intravascular Coagulation""[All Fields]) OR ((""coagulation""[All Fields] AND ""intravascular""[All Fields]) AND ""disseminated""[All Fields])) )) OR ((((""Disseminated Intravascular Coagulation""[MeSH Terms] OR ((""disseminated""[All Fields] AND ""intravascular""[All Fields]) AND ""coagulation""[All Fields])) OR ""Disseminated Intravascular Coagulation""[All Fields]) OR ((""coagulations""[All Fields] AND ""intravascular""[All Fields]) AND ""disseminated""[All Fields])) )) OR ((((""Disseminated Intravascular Coagulation""[MeSH Terms] OR ((""disseminated""[All Fields] AND ""intravascular""[All Fields]) AND ""coagulation""[All Fields])) OR ""Disseminated Intravascular Coagulation""[All Fields]) OR ((""disseminated""[All Fields] AND ""coagulations""[All Fields]) AND ""intravascular""[All Fields])) )) OR ((((""Disseminated Intravascular Coagulation""[MeSH Terms] OR ((""disseminated""[All Fields] AND ""intravascular""[All Fields]) AND ""coagulation""[All Fields])) OR ""Disseminated Intravascular Coagulation""[All Fields]) OR ((""intravascular""[All Fields] AND ""disseminated""[All Fields]) AND ""coagulations""[All Fields])) )) OR ""intravascular coagulation disseminated""[All Fields]) OR ""Intravascular Disseminated Coagulation""[All Fields]) OR ""coagulation disseminated intravascular""[All Fields]) OR ((((""Disseminated Intravascular Coagulation""[MeSH Terms] OR ((""disseminated""[All Fields] AND ""intravascular""[All Fields]) AND ""coagulation""[All Fields])) OR ""Disseminated Intravascular Coagulation""[All Fields]) OR ((""coagulations""[All Fields] AND ""disseminated""[All Fields]) AND ""intravascular""[All Fields])) )) OR ""Disseminated Intravascular Coagulations""[All Fields]) OR ((((""Disseminated Intravascular Coagulation""[MeSH Terms] OR ((""disseminated""[All Fields] AND ""intravascular""[All Fields]) AND ""coagulation""[All Fields])) OR ""Disseminated Intravascular Coagulation""[All Fields]) OR ((""intravascular""[All Fields] AND ""coagulations""[All Fields]) AND ""disseminated""[All Fields])) )) OR ""Consumption Coagulopathy""[All Fields]) OR ((((""Disseminated Intravascular Coagulation""[MeSH Terms] OR ((""disseminated""[All Fields] AND ""intravascular""[All Fields]) AND ""coagulation""[All Fields])) OR ""Disseminated Intravascular Coagulation""[All Fields]) OR (""coagulopathies""[All Fields] AND ""consumption""[All Fields])) )) OR ""coagulopathy consumption""[All Fields]) OR ""Consumption Coagulopathies""[All Fields]) OR ""Disseminated Intravascular Coagulation""[MeSH Terms]) OR (""sepsis""[MeSH Terms] OR (((((((((((((((((((""sepsis""[MeSH Terms] OR ""sepsis""[All Fields]) OR (((""sepsis""[MeSH Terms] OR ""sepsis""[All Fields]) OR ""pyaemia""[All Fields]) OR ""pyemia""[All Fields])) OR ((""sepsis""[MeSH Terms] OR ""sepsis""[All Fields]) OR ""pyemias""[All Fields])) OR ((""sepsis""[MeSH Terms] OR ""sepsis""[All Fields]) OR ""pyohemia""[All Fields])) OR ((""sepsis""[MeSH Terms] OR ""sepsis""[All Fields]) )) OR (((""sepsis""[MeSH Terms] OR ""sepsis""[All Fields]) OR ""pyaemia""[All Fields]) OR ""pyemia""[All Fields])) OR ((""sepsis""[MeSH Terms] OR ""sepsis""[All Fields]) )) OR (((((""sepsis""[MeSH Terms] OR ""sepsis""[All Fields]) OR ""septicaemias""[All Fields]) OR ""septicemias""[All Fields]) OR ""septicaemia""[All Fields]) OR ""septicemia""[All Fields])) OR (((((""sepsis""[MeSH Terms] OR ""sepsis""[All Fields]) OR ""septicaemias""[All Fields]) OR ""septicemias""[All Fields]) OR ""septicaemia""[All Fields]) OR ""septicemia""[All Fields])) OR ""poisoning blood""[All Fields]) OR ""Blood Poisoning""[All Fields]) OR ""Blood Poisonings""[All Fields]) OR (((""sepsis""[MeSH Terms] OR ""sepsis""[All Fields]) OR (""poisonings""[All Fields] AND ""blood""[All Fields])) )) OR ""Severe Sepsis""[All Fields]) OR ""sepsis severe""[All Fields]) OR ""septic shock""[All Fields]) OR ""shock septic""[All Fields]) OR ""bactermia""[All Fields]) OR ""systemic inflammatory response syndrome""[All Fields]))) AND (""heparin""[MeSH Terms] OR (((((((((""Unfractionated Heparin""[All Fields] OR ""heparin unfractionated""[All Fields]) OR ""Heparinic Acid""[All Fields]) OR ((""heparin""[MeSH Terms] OR ""heparin""[All Fields]) OR ""liquaemin""[All Fields])) OR ""Sodium Heparin""[All Fields]) OR ""Heparin Sodium""[All Fields]) OR ""Heparin Sodium""[All Fields]) OR (((""heparin""[MeSH Terms] OR ""heparin""[All Fields]) OR (""alpha""[All Fields] AND ""heparin""[All Fields])) OR ""alpha Heparin""[All Fields])) OR ""alpha Heparin""[All Fields]) OR (((((((((((((((""heparin""[MeSH Terms] OR ""heparin""[All Fields]) OR ""heparine""[All Fields]) OR ""heparins""[All Fields]) OR ""heparin s""[All Fields]) OR ""heparinate""[All Fields]) OR ""heparinated""[All Fields]) OR ""heparines""[All Fields]) OR ""heparinic""[All Fields]) OR ""heparinisation""[All Fields]) OR ""heparinised""[All Fields]) ) OR ""heparinization""[All Fields]) OR ""heparinize""[All Fields]) OR ""heparinized""[All Fields]) OR ""heparinizing""[All Fields])))","3,663" |
| #12 | """randomized controlled trails"" or ""randomized controlled trail"" or RCT or randomized or "" clinical trail""",,,"(((""randomized controlled trails""[All Fields] OR ""randomized controlled trail""[All Fields]) OR ""RCT""[All Fields]) OR ((((((((((((((((""random allocation""[MeSH Terms] OR (""random""[All Fields] AND ""allocation""[All Fields])) OR ""random allocation""[All Fields]) OR ""random""[All Fields]) OR ""randomization""[All Fields]) OR ""randomized""[All Fields]) OR ""randomisation""[All Fields]) OR ""randomisations""[All Fields]) OR ""randomise""[All Fields]) OR ""randomised""[All Fields]) OR ""randomising""[All Fields]) OR ""randomizations""[All Fields]) OR ""randomize""[All Fields]) OR ""randomizes""[All Fields]) OR ""randomizing""[All Fields]) OR ""randomness""[All Fields]) OR ""randoms""[All Fields])) OR ""clinical trail""[All Fields]","1,188,769" |
| #13 | "(""randomized controlled trails"" or ""randomized controlled trail"" or RCT or randomized or "" clinical trail"") AND ((((""Disseminated Coagulation, Intravascular"" or ""Coagulation, Intravascular Disseminated"" or ""Coagulations, Intravascular Disseminated"" or ""Disseminated Coagulations, Intravascular"" or ""Intravascular Disseminated Coagulations"" or ""Intravascular Coagulation, Disseminated"" or ""Intravascular Disseminated Coagulation"" or ""Coagulation, Disseminated Intravascular"" or ""Coagulations, Disseminated Intravascular"" or ""Disseminated Intravascular Coagulations"" or ""Intravascular Coagulations, Disseminated"" or ""Consumption Coagulopathy"" or ""Coagulopathies, Consumption"" or ""Coagulopathy, Consumption"" or ""Consumption Coagulopathies"") OR (""Disseminated Intravascular Coagulation"" [MeSH Terms])) OR ((sepsis[MeSH Terms]) OR (sepsis or Pyemia or Pyemias or Pyohemia or Pyohemias or Pyaemia or Pyaemias or Septicemia or Septicemias or ""Poisoning, Blood"" or ""Blood Poisoning"" or ""Blood Poisonings"" or ""Poisonings, Blood"" or ""Severe Sepsis"" or ""Sepsis, Severe"" or ""septic shock"" or "" shock, septic"" or bactermia or "" systemic inflammatory response syndrome""))) AND ((heparin[MeSH Terms]) OR (""Unfractionated Heparin"" or ""Heparin, Unfractionated"" or ""Heparinic Acid"" or Liquaemin or ""Sodium Heparin"" or ""Heparin, Sodium"" or ""Heparin Sodium"" or alpha-Heparin or ""alpha Heparin"" or heparin)))",,,"((((""randomized controlled trails""[All Fields] OR ""randomized controlled trail""[All Fields]) OR ""RCT""[All Fields]) OR ((((((((((((((((""random allocation""[MeSH Terms] OR (""random""[All Fields] AND ""allocation""[All Fields])) OR ""random allocation""[All Fields]) OR ""random""[All Fields]) OR ""randomization""[All Fields]) OR ""randomized""[All Fields]) OR ""randomisation""[All Fields]) OR ""randomisations""[All Fields]) OR ""randomise""[All Fields]) OR ""randomised""[All Fields]) OR ""randomising""[All Fields]) OR ""randomizations""[All Fields]) OR ""randomize""[All Fields]) OR ""randomizes""[All Fields]) OR ""randomizing""[All Fields]) OR ""randomness""[All Fields]) OR ""randoms""[All Fields])) OR ""clinical trail""[All Fields]) AND (((((((((((((((((((((""Disseminated Intravascular Coagulation""[MeSH Terms] OR ((""disseminated""[All Fields] AND ""intravascular""[All Fields]) AND ""coagulation""[All Fields])) OR ""Disseminated Intravascular Coagulation""[All Fields]) OR ((""disseminated""[All Fields] AND ""coagulation""[All Fields]) AND ""intravascular""[All Fields])) ) OR ((((""Disseminated Intravascular Coagulation""[MeSH Terms] OR ((""disseminated""[All Fields] AND ""intravascular""[All Fields]) AND ""coagulation""[All Fields])) OR ""Disseminated Intravascular Coagulation""[All Fields]) OR ((""coagulation""[All Fields] AND ""intravascular""[All Fields]) AND ""disseminated""[All Fields])) )) OR ((((""Disseminated Intravascular Coagulation""[MeSH Terms] OR ((""disseminated""[All Fields] AND ""intravascular""[All Fields]) AND ""coagulation""[All Fields])) OR ""Disseminated Intravascular Coagulation""[All Fields]) OR ((""coagulations""[All Fields] AND ""intravascular""[All Fields]) AND ""disseminated""[All Fields])) )) OR ((((""Disseminated Intravascular Coagulation""[MeSH Terms] OR ((""disseminated""[All Fields] AND ""intravascular""[All Fields]) AND ""coagulation""[All Fields])) OR ""Disseminated Intravascular Coagulation""[All Fields]) OR ((""disseminated""[All Fields] AND ""coagulations""[All Fields]) AND ""intravascular""[All Fields])) )) OR ((((""Disseminated Intravascular Coagulation""[MeSH Terms] OR ((""disseminated""[All Fields] AND ""intravascular""[All Fields]) AND ""coagulation""[All Fields])) OR ""Disseminated Intravascular Coagulation""[All Fields]) OR ((""intravascular""[All Fields] AND ""disseminated""[All Fields]) AND ""coagulations""[All Fields])) )) OR ""intravascular coagulation disseminated""[All Fields]) OR ""Intravascular Disseminated Coagulation""[All Fields]) OR ""coagulation disseminated intravascular""[All Fields]) OR ((((""Disseminated Intravascular Coagulation""[MeSH Terms] OR ((""disseminated""[All Fields] AND ""intravascular""[All Fields]) AND ""coagulation""[All Fields])) OR ""Disseminated Intravascular Coagulation""[All Fields]) OR ((""coagulations""[All Fields] AND ""disseminated""[All Fields]) AND ""intravascular""[All Fields])) )) OR ""Disseminated Intravascular Coagulations""[All Fields]) OR ((((""Disseminated Intravascular Coagulation""[MeSH Terms] OR ((""disseminated""[All Fields] AND ""intravascular""[All Fields]) AND ""coagulation""[All Fields])) OR ""Disseminated Intravascular Coagulation""[All Fields]) OR ((""intravascular""[All Fields] AND ""coagulations""[All Fields]) AND ""disseminated""[All Fields])) )) OR ""Consumption Coagulopathy""[All Fields]) OR ((((""Disseminated Intravascular Coagulation""[MeSH Terms] OR ((""disseminated""[All Fields] AND ""intravascular""[All Fields]) AND ""coagulation""[All Fields])) OR ""Disseminated Intravascular Coagulation""[All Fields]) OR (""coagulopathies""[All Fields] AND ""consumption""[All Fields])) )) OR ""coagulopathy consumption""[All Fields]) OR ""Consumption Coagulopathies""[All Fields]) OR ""Disseminated Intravascular Coagulation""[MeSH Terms]) OR (""sepsis""[MeSH Terms] OR (((((((((((((((((((""sepsis""[MeSH Terms] OR ""sepsis""[All Fields]) OR (((""sepsis""[MeSH Terms] OR ""sepsis""[All Fields]) OR ""pyaemia""[All Fields]) OR ""pyemia""[All Fields])) OR ((""sepsis""[MeSH Terms] OR ""sepsis""[All Fields]) OR ""pyemias""[All Fields])) OR ((""sepsis""[MeSH Terms] OR ""sepsis""[All Fields]) OR ""pyohemia""[All Fields])) OR ((""sepsis""[MeSH Terms] OR ""sepsis""[All Fields]) )) OR (((""sepsis""[MeSH Terms] OR ""sepsis""[All Fields]) OR ""pyaemia""[All Fields]) OR ""pyemia""[All Fields])) OR ((""sepsis""[MeSH Terms] OR ""sepsis""[All Fields]) )) OR (((((""sepsis""[MeSH Terms] OR ""sepsis""[All Fields]) OR ""septicaemias""[All Fields]) OR ""septicemias""[All Fields]) OR ""septicaemia""[All Fields]) OR ""septicemia""[All Fields])) OR (((((""sepsis""[MeSH Terms] OR ""sepsis""[All Fields]) OR ""septicaemias""[All Fields]) OR ""septicemias""[All Fields]) OR ""septicaemia""[All Fields]) OR ""septicemia""[All Fields])) OR ""poisoning blood""[All Fields]) OR ""Blood Poisoning""[All Fields]) OR ""Blood Poisonings""[All Fields]) OR (((""sepsis""[MeSH Terms] OR ""sepsis""[All Fields]) OR (""poisonings""[All Fields] AND ""blood""[All Fields])) )) OR ""Severe Sepsis""[All Fields]) OR ""sepsis severe""[All Fields]) OR ""septic shock""[All Fields]) OR ""shock septic""[All Fields]) OR ""bactermia""[All Fields]) OR ""systemic inflammatory response syndrome""[All Fields]))) AND (""heparin""[MeSH Terms] OR (((((((((""Unfractionated Heparin""[All Fields] OR ""heparin unfractionated""[All Fields]) OR ""Heparinic Acid""[All Fields]) OR ((""heparin""[MeSH Terms] OR ""heparin""[All Fields]) OR ""liquaemin""[All Fields])) OR ""Sodium Heparin""[All Fields]) OR ""Heparin Sodium""[All Fields]) OR ""Heparin Sodium""[All Fields]) OR (((""heparin""[MeSH Terms] OR ""heparin""[All Fields]) OR (""alpha""[All Fields] AND ""heparin""[All Fields])) OR ""alpha Heparin""[All Fields])) OR ""alpha Heparin""[All Fields]) OR (((((((((((((((""heparin""[MeSH Terms] OR ""heparin""[All Fields]) OR ""heparine""[All Fields]) OR ""heparins""[All Fields]) OR ""heparin s""[All Fields]) OR ""heparinate""[All Fields]) OR ""heparinated""[All Fields]) OR ""heparines""[All Fields]) OR ""heparinic""[All Fields]) OR ""heparinisation""[All Fields]) OR ""heparinised""[All Fields]) ) OR ""heparinization""[All Fields]) OR ""heparinize""[All Fields]) OR ""heparinized""[All Fields]) OR ""heparinizing""[All Fields]))))",290 |
