# Supplementary material for: Unfractionated heparin improves the clinical efficacy in adult sepsis patients: a systematic review and meta-analysis
Source: BMC Anesthesiol. 2022 Jan 21;22:28. doi: 10.1186/s12871-021-01545-w (PMC8777179; doi:10.1186/s12871-021-01545-w)
Supplement: Supplementary file 10 — Additional file 10 : Table S2. Excluded literature information. [file 12871_2021_1545_MOESM10_ESM.docx]

Additional file 10 Excluded literature

| Title | Year | Reason |
| --- | --- | --- |
| **Chinese articles**  Evaluation of clinical effects off low-dose heparin therapy for sepsis | 2007 | No relevant outcomes |
| The clinical study on acute lung injury or acute respiratory distress syndrome with sepsis treated with continuous low dose heparin | 2010 | Quasi-randomized controlled trials |
| The clinical care of 119 patients with sepsis treated with continuous low dose heparin | 2010 | Quasi-randomized controlled trials |
| Clinical efficacy of continuous low-dose heparin in the treatment of severe sepsis | 2011 | Quasi-randomized controlled trials |
| The clinical observation of early heparinization in the treatment of sepsis | 2013 | Study objective did not match |
| The clinical observation of low dose unfractionated heparin in the auxiliary effect of sepsis. In: Chinese medical association of emergency medicine | 2014 | The results were not complete |
| Clinical efficacy of low dose of heparin in treatment of patients with sepsis and its influence on coagulation indexes | 2015 | Study objective did not match |
| Clinical efficacy of low dose of heparin in treatment of patients with sepsis and its influence on coagulation function and platelet activation | 2015 | No relevant outcomes |
| The effect of anticoagulant therapy on coagulation and inflammation markers in sepsis patients and its significance | 2015 | Study objective did not match |
| Clinical curative effect of low dose heparin combined with Xuebijing in the treatment of sepsis | 2015 | No relevant outcomes |
| The Effect of Low-dose Heparin on Peripheral Inflammatory Cytokines and Cell Adhesion Molecules of Patients with Sepsis | 2016 | No relevant outcomes |
| The effect of heparin therapy on lung and renal function in patients with severe sepsis | 2017 | No relevant outcomes |
| **English articles** |  |  |
| The influence of heparin on intravenous infusions: a prospective study | 1980 | Study objective did not match |
| Use of antithrombin III concentrates. Beitrage zur Infusionstherapie [Contributions to infusion therapy] | 1989 | There were too small amounts of participants and failed to find full-text |
| Sepsis: a collaborative study on the treatment of coagulation disturbances with antithrombin III or heparin | 1997 | Failed to find the full-text |
| Subcutaneous heparin prophylaxis significantly reduces the incidence of venous thromboembolic events in the critically ill | 1999 | Study objective did not match |
| Heparin blunts endotoxin-induced coagulation activation | 1999 | Study objective did not match |
| High-dose antithrombin III in severe sepsis: a randomized controlled trial | 2001 | Quasi-randomized controlled trials |
| Evaluation of anti-inflammatory and antiadhesive effects of heparins in human endotoxemia | 2003 | Non randomized controlled trials |
| Phase III Clinical Study on ART-123 in patients with disseminated intravascular coagulation (DIC) directly caused by malignant hematopoietic tumors or infections | 2006 | Study objective did not match |
| A randomized clinical trial of unfractionated heparin for treatment of sepsis (the HETRASE study): design and rationale. | 2006 | Protocol |
| Comprehensive safety analysis of concomitant drotrecogin alfa (activated) and prophylactic heparin use in patients with severe sepsis | 2009 | Secondary analysis |
| Venous thromboembolism in critically ill patients. Observations from a randomized trial in sepsis | 2009 | Study objective did not match |
| Thrombomodulin alfa in the treatment of infection patients complicated by disseminated intra-vascular coagulation: Sub-analysis from the phase trial | 2011 | Study objective did not match |
| Dalteparin did not differ from unfractionated heparin for reducing proximal DVT in critically ill patients | 2011 | Study objective did not match |
| Dalteparin versus unfractionated heparin in critically ill patients | 2011 | Study objective did not match |
| PROphylaxis for Thrombo-Embolism in Critical Care Trial protocol and analysis plan | 2011 | Study objective did not match |
| Nct: Heparin Anticoagulation to Improve Outcomes in Septic Shock: the HALO Pilot | 2012 | Protocol |
| A prospective clinical study of low-dose heparin as an intervention treatment of Sepsis accompanied with pre-DIC | 2012 | Study objective did not match |
| Nct: Impact of Low Dose Unfractionated Heparin Treatment on Inflammation in Sepsis | 2014 | Protocol |
| Heparin Effect in sepsis | 2015 | Study objective did not match |
| Failure of anticoagulant thromboprophylaxis: risk factors in medical-surgical critically ill patients | 2015 | Study objective did not match |
| Efficacy and Safety of Unfractionated Heparin on Severe Sepsis With Suspected Disseminated Intravascular Coagulation | 2015 | Protocol |
| Low-dose heparin in critically Ill patients undergoing extracorporeal membrane oxygenation-the help-ECMO pilot randomised controlled trial | 2016 | Study objective did not match |
| Low-dose heparin versus full systemic anticoagulation in critically ill patients undergoing extracorporeal membrane oxygenation: the HELP-ECMO pilot randnomised controlled study | 2017 | Study objective did not match |
| Benefit of anti-coagulant therapy in patients with septic shock | 2019 | Clinical study is ongoing |
