# Supplementary material for: Unfractionated heparin improves the clinical efficacy in adult sepsis patients: a systematic review and meta-analysis
Source: BMC Anesthesiol. 2022 Jan 21;22:28. doi: 10.1186/s12871-021-01545-w (PMC8777179; doi:10.1186/s12871-021-01545-w)
Supplement: Supplementary file 11 — Additional file 11 : Table S3. Evidence assessment. [file 12871_2021_1545_MOESM11_ESM.docx]

Additional file 11

| Outcomes | Studies | Participants | Quality Assessment | | | | | | Quality of evidence |
| --- | --- | --- | --- | --- | --- | --- | --- | --- | --- |
|  |  |  | Study design | Risk of bias | Inconsistency | Indirectness | Imprecision | Publication bias |  |
| 28 d mortality | 13 | 2417 | RCT | No serious risk of bias | NO | NO | NO | Basic symmetry | High quality |
| PLT | 8 | 539 | RCT | No serious risk of bias | NO | NO | NO | Basic symmetry | High quality |
| PT | 8 | 539 | RCT | No serious risk of bias | NO | NO | NO | Asymmetry | Low quality |
| APTT | 8 | 539 | RCT | No serious risk of bias | NO | NO | NO | Asymmetry | Low quality |
| MODS incidence | 4 | 346 | RCT | No serious risk of bias | NO | NO | NO | Insufficient experimental data | Moderate quality |
| LOS | 5 | 301 | RCT | No serious risk of bias | NO | NO | NO | Insufficient experimental data | Moderate quality |
| Duration of Ventilation | 4 | 318 | RCT | No serious risk of bias | NO | NO | NO | Insufficient experimental data | Moderate quality |
| Bleeding complication | 4 | 1962 | RCT | No serious risk of bias | NO | NO | NO | Insufficient experimental data | Moderate quality |
